# Psychosomatic problems among medical students: a myth or reality?

**DOI:** 10.1186/s13033-016-0105-3

**Published:** 2016-11-24

**Authors:** J. M. Chinawa, Ada R. C. Nwokocha, Pius C. Manyike, Awoere Tamunosiki Chinawa, Elias C. Aniwada, Appolos Chidi Ndukuba

**Affiliations:** 1 College of Medicine, Department of Pediatrics, University of Nigeria Teaching Hospital (UNTH), Ituku Ozalla, Enugu, Nigeria; 2 University of Nigeria Enugu Campus, Enugu, Nigeria; 3 College of Medicine, Department of Pediatrics, Federal Teaching Hospital Abakiliki, Abakiliki, Nigeria; 4 College of Medicine, Department of Community Medicine, Enugu State University Teaching Hospital, Enugu, Enugu State Nigeria; 5 College of Medicine, Department of Community Medicine, University of Nigeria Teaching Hospital (UNTH), Ituku Ozalla, Enugu, Nigeria; 6 College of Medicine, Department of Psychological Medicine, University of Nigeria Teaching Hospital (UNTH), Ituku Ozalla, Enugu, Nigeria

**Keywords:** Psychosomatic disorder, Medical students, Nigeria

## Abstract

**Background:**

Medical students are exposed to stress and this can predispose them to psychological and behavioral consequences.

**Methods:**

Psychosomatic disorders were investigated among 385 medical students from two teaching hospitals using a stratified random sampling. The Enugu somatization Scale (ESS) was used to evaluate for presence of somatization in the participants. Statistical analysis was done with the Statistical Package for Social Sciences (SPPS) version 19 (Chicago IL).

**Results:**

A total of 385 medical students with a calculated mean age of 23.55 ± 3.33 years were recruited in this study. The prevalence of psychosomatic disorder was 55 (14.3%) with prevalence among males 33 (14.2%) and among females 22 (14.4%). Based on features, 44 (11.4%) had head features while 30 (7.8%) had body features of psychosomatic disorder respectively. Similar proportion of both males and females (about 14% each) had psychosomatic disorder. There was no statistically significant difference ($\chi^{2}$ = 0.002, p = 0.966). Students aged 24 years and below had similar proportion of psychosomatic disorder 38 (14.3%) with those aged over 24 years 17 (14.2%). The difference was not statistically significant ($\chi^{2}$ = 0.002, p = 0.964). Students from lower social class had lower proportion of psychosomatic disorder (10.6%) when compared to middle (17.2%) and upper (15.2%). The difference was equally not statistically significant ($\chi^{2}$ = 1.759, p = 0.415). Male students had similar likelihood of psychosomatic disorder with females (OR 1.01, 95% CI 0.56−1.82). Those had belong to middle socio-economic class were about 1.2 times (AOR 1.15, 95% CI 0.54−2.45) and lower socio-economic class about 0.6 times (AOR 0.66, 95% CI 0.31−1.37) likely to have psychosomatic disorder than those from upper socio-economic class.

**Conclusions:**

Psychosomatic disorders constitute an emerging mental health problem among medical students in Nigerian Universities. This can pose a major mental health problem if neglected.

**Electronic supplementary material:**

The online version of this article (doi:10.1186/s13033-016-0105-3) contains supplementary material, which is available to authorized users.

## Background

Psychosomatization is defined as multiple, recurrent and frequently changing physical symptoms usually present for at least two years before the patient is referred to a psychiatrist [1]. In the Diagnostic and Statistical manual of Mental disorders—5th edition (DSM-5), however, the condition has been renamed somatic symptom disorder (SSD) which includes somatization disorder, hypochondriasis, pain disorder, and undifferentiated somatoform disorder [2].

Psychosomatic disorders (PSD) have been attributed to inappropriate activation of the autonomous nervous apparatus, the endocrine and the immune systems (defense structures and cells) [3].

It is well documented fact that more than 70% of medical students present with different symptoms suggestive of psycho-somatization [4]. More recent research, however, indicates that the phenomenon could have been exaggerated [4].

Although psychosomatic disorders among medical school are often neglected, In fact over the years, this disorder was trivialized and even given names as medical students’ disease or “medical studentitis” [4]. However it is known that this health problems triggers anxiety among students.

Studies done about two decades ago showed that medical students are more likely to have psychosomatic disorders than law students [5]. However, Moss et al. [5] noted that medical students who are diagnosed as having psychosomatic disorders could be a misnomer since it could be an anxiety issues stemming from stressful medical curriculum [5].

Notwithstanding, medical students who suffer from these disorders may not be malingering as the pain and other problems they experience could be real [6]. This disorder can place significant burden on the healthcare delivery system, which can result in heavy utilization of resources through repeated hospitalizations [7]. Moreover, psychosomatic disorders among medical students could be associated with poor school performance and attendance [8].

Delay in diagnosing PSDs should be avoided as much as possible. This is because failure to make accurate and timely diagnosis often results in multiple referrals, repeated unnecessary diagnostic tests, unjustified and potentially harmful treatments including medication trials and even surgeries, and the perpetuation of the belief of underlying organic illness [9].

In the contrary, Moss et al. [5] opined that understanding medical students’ disease (psychosomatic disorder) as a normal process may be used to educate medical students when they enroll in the medical school, so as to reduce the distress associated with the condition.

This study aims to determine the pattern of presentation of PSDs and its socioeconomic determinants among medical students in South east Nigeria. In this part of the country, there are very few studies on PSDs among medical students in tertiary institutions and much has not been documented on this topic due to neglect and misdiagnosis.

## Methods

### Setting

The study was carried out among medical students in Enugu and Ebonyi State which is located in South east Nigeria.

### Instrument used

A pretested self—administered questionnaire from the Enugu Somatization Scale (ESS) developed by Ebigbo [10] was used for this study. This instrument was developed bearing in mind the shortcomings or uncertainties that accompany the use of western methods of assessment techniques in our setting. The scale is a 65-item scale two sub-scales [10]. The first is the HEAD subscale, captured by items 1–23, while the BODY subscale is captured by items 24–65. The ESS has a dichotomous response options which are YES and NO. It has been cross-validated with Neurotic Illness Questionnaire (NIQ) in India where the ESS was found to correlate significantly with NIQ [6]. A score of 1 is assigned to any Yes while a score of 0 is assigned to any NO response. See Additional file 1.

### Study population

Four hundred and sixty-one medical students who met inclusion criteria were consecutively recruited between June and October, 2014 from two medical schools from south east Nigeria. The medical schools were selected by listing all the accredited medical schools in southeast Nigeria and then two medical schools were selected using simple random sampling. Four hundred and sixty questionnaires were administered to those that gave consent but 385 that gave consent and responded well were studied. This gave a response rate of 83.7%.

Socioeconomic class was ascertained using a recommended method, modified by Oyedeji [11]. Parents’ occupations and highest level of education were assigned a score from 1 (highest) to 5 (lowest). The mean score for both parents provided a score for social class that fell within the 1–5 range. Those with a mean score <2 were further sub-classified into upper class, while those with a mean score >2 were sub-classified into a lower social class. For the occupation score, those in the upper social class included parents whose occupations included positions as senior public officers, large-scale traders, large-scale farmers, and professionals. Lower class occupations included artisans, primary school teachers, peasant farmers, laborers, and the unemployed. For the education score, those with a Ph.D., Master’s degree, Bachelor’s degree, or higher national diploma were categorized as upper class. Those with an ordinary national diploma, national certificate of education, technical education, grade II teachers’ certificate, junior or senior secondary school certificate, primary school certificate, or those with no formal education were classified as lower social class [11]. Medical students who gave consent were included in this study while those without consent were excluded from the study.

### Ethical consideration

Consent was specifically obtained from medical students from both Universities while Ethical clearance for the study was obtained from the Research and Ethical Committee of the University of Nigeria Teaching Hospital Ituku Ozalla.

### Data analysis

Statistical analysis was with Statistical Package for Social Sciences (SPPS) version 19 (Chicago IL). Chi square test was used to test for statistical association of categorical variables. Age was not normally distributed. All reported p values are 2-sided and values <0.05 were assumed as significant.

## Results

A total of 385 medical students with a calculated mean age of 23.55 ± 3.33 years were recruited in this study. Two hundred and seventeen 217 (56.4%) and 104 (27.0%) belonged to upper and lower social class respectively. Over 95% of them are still not married. Higher proportion of respondents were aged 24 years and below. The mean and standard deviation of age of respondents for males and females were 23.1 (3.2) and 24.9 (4.0) (Table 1).

**Table 1:** Socio-demographics of respondents

Variables	Frequency (n = 385)	Percentage
Age in categories (years)		
≤24	265	68.8
>24	120	31.2
Mean (SD) for females	23.1 (3.2)	
Mean (SD) for males	24.9 (4.0)	
Sex		
Male	232	60.3
Female	153	39.7
Marital status		
Single	368	95.6
Married	17	4.4
Social class		
Upper	217	56.4
Middle	64	16.6
Lower	104	27.0

Table 2 shows that prevalence of psychosomatic disorder was 55 (14.3%) with prevalence among males 33 (14.2%) and among females 22 (14.4%). Based on features, 44 (11.4%) had head features while 30 (7.8%) had body features of psychosomatic disorder respectively.

**Table 2:** Prevalence of psychosomatic disorder; head and body features

Variables	n = 385	
	Presence freq (%)	Absence freq (%)
Psychosomatic disorder		
General	55 (14.3)	330 (85.7)
Males	33 (14.2)	199 (85.8)
Females	22 (14.4)	131 (85.6)
Head features	44 (11.4)	341 (88.6)
Body features	30 (7.8)	355 (92.2)

Table 3 shows that similar proportion of both males and females (about 14% each) had psychosomatic disorder. There was no statistically significant difference ($\chi^{2}$ = 0.002, p = 0.966). Students aged 24 years and below had similar proportion of psychosomatic disorder 38 (1014.3%) with those aged over 24 years 17 (14.2%).The difference was not statistically significant ($\chi^{2}$ = 0.002, p = 0.964). Students from lower social class had lower proportion of psychosomatic disorder (10.6%) when compared to middle (17.2%) and upper (15.2%). The difference was equally not statistically significant ($\chi^{2}$ = 1.759, p = 0.415).

**Table 3:** Relationship between socio-demographics and psychosomatic disorder

Variables	n = 385		Test statistics $\chi^{2}$	p value
	Presence	Absence		
	n (%)	n (%)		
Age in categories (years)				
≤24	38 (14.3)	227 (85.7)	0.002	0.964
>24	17 (14.2)	103 (85.8)		
Sex				
Male	33 (14.2)	199 (85.8)	*0.002*	*0.966*
Female	22 (14.4)	131 (85.6)		
Social class				
Upper	33 (15.2)	184 (84.8)		
Middle	11 (17.2)	53 (82.8)	*1.759*	*0.415*
Lower	11 (10.6)	93 (89.4)		

Italic values indicate significance of p values (p < 0.05)

Table 4 shows that students aged above 24 years had similar likelihood of psychosomatic disorder with those aged 24 years and below (OR 1.03, 95% CI 0.55−1.93). Male students had similar likelihood of psychosomatic disorder with females (OR 1.01, 95% CI 0.56−1.82). Those had belong to middle socio-economic class were about 1.2 times (AOR 1.15, 95% CI 0.54−2.45) and lower socio-economic class about 0.6 times (AOR 0.66, 95% CI 0.31−1.37) likely to have psychosomatic disorder than those from upper socio-economic class.

**Table 4:** Predictors of psychosomatic disorder (logistic regression)

Variables	OR	Sig	95% CI for OR	
			Lower	Upper
Age in categories (years)				
≤24				
>24	1.031	0.924	0.549	1.935
Sex				
Female				
Male	1.007	0.982	0.557	1.820
Social class				
Upper				
Middle	1.153	0.711	0.543	2.448
Lower	0.656	0.261	0.314	1.369

## Discussion

This study has shown that psychosomatic disorders do exist among medical students in two south east Nigerian universities with a prevalence rate of 14.3%. This is similar to the prevalence of 17.0% documented by Susanne [12] but higher than that seen in other studies [13]. For instance, Firth et al. [14] noted a prevalence of psychosomatic disorder to be 31.2% among medical students, and another study carried out on new medical graduates showed a prevalence of 26% [15].

Azuri et al. [16] noted a significant prevalence of psychosomatic disorder when enrolling for clinical posting with a significant decrease later on. Furthermore, in one Australian study, the prevalence of somatization among Australian general practitioners revealed a higher prevalence of 18.6% which is more than that seen in our study [17]. In addition, Cande et al. [18], noted that 30% of medical students reported a history of psychosomatization and only 22% of them consulted a medical specialist.

In the general population, the prevalence of PSD using DSM-IV was found to be low (0.05%), while it was higher (0.58%) using less restrictive diagnostic criteria than DSM-IV [19]. This prevalence is lower when compared to that of medical students obtained in this study.

The reason for the disparity in prevalence rates is probably due to the differences in ethno-cultural construct. Comparison of prevalence studies for psychosomatic disorders is difficult, due to a lack of homogeneity [20]. Furthermore, some studies focus on somatoform disorders alone, while others include all forms of mental illness [21].

Different psychiatric disorders are usually under- recognized yet they are common and treatable among medical students [22]. Various studies have shown that medical students are subjected to considerable mental distress during their training and this predicts psychosomatic problems in physicians later in life. This disorder in the individual doctor might negatively affect patient care. Normally, physicians do not seek the kind of professional help for themselves, as they would provide for their patients. Medical students seem to adopt a similar pattern of behavior.

However, some authors postulated that Psychosomatic disorder as been groundless and suggested that the symptoms of psychosomatization are imagined with undeserved importance being placed on bodily sensations that do not clinically warrant either medical attention or the degree of anxiety that they have provoked [23].

We noted almost the same proportion of participants with head and body symptoms. This could reflect the poly-symptomatic nature of the condition [24].

In the general population, psychosomatic disorders can present with arm, neck and shoulder symptoms [25]. In 2003, 12-month prevalence in the Netherlands showed 31.4% of subjects had neck symptoms, 30.3% shoulder symptoms and 17.5% had wrist symptoms. It is also noted among Nigerian undergraduates where a high prevalence of shoulder symptoms was most common [26]. In Australia, musculoskeletal features were a widespread problem for university students [27]. In addition, in Saudi Arabia, students complained of headache and fatigue as major PSD symptoms [28].

There is no gender difference among medical students with psychosomatic disorder as seen in this study. There is a changing trend in gender distribution of psychiatric illness of which PSD is not exempted. Studies have reported an ablation of gender differences in most psychiatric disorder [22–24]. However, some studies conducted in Germany, revealed a highly significant increase in prevalence among female medical students and an increase in the female prevalence in the general population [29, 30]. The difference in this prevalence in gender could not be ascertained.

Though students from lower middle and higher socio economic class had a higher proportion of psychosomatic disorder when compared to those with a lower class, the difference was not statistically significant.

Iheme et al. [31] noted a higher episode of somatization disorder among the upper socioeconomic class. Again differences in ethno-cultural construct and differences geographic location can explain this socioeconomic difference.

## Conclusion

Psychosomatic disorders constitute an emerging mental health problem among medical students in Nigerian universities. Although they appear overlooked, the prevalence rate may be rising.

## Limitation

The limitation of this study lies in the fact that only teaching hospitals from south east Nigeria were recruited for the study. Furthermore, this is a cross sectional study with an attrition rate. A wider study in the entire nation’s teaching hospital, though tasking, will give a better picture of the prevalence.

## Additional file

**Additional file 1.** Norms for the Enugu summarization scale.

## Abbreviations

ESS: the Enugu somatization scale
SPPS: statistical package for social sciences
SSD: somatic symptom disorder
PSD: psychosomatic disorders
NIQ: neurotic illness questionnaire
