# Supplementary material for: Psychosomatic problems among medical students: a myth or reality?
Source: Int J Ment Health Syst. 2016 Nov 24;10:72. doi: 10.1186/s13033-016-0105-3 (PMC5123430; doi:10.1186/s13033-016-0105-3)
Supplement: Supplementary file 1 — Additional file 1. Norms for the Enugu summarization scale. [file 13033_2016_105_MOESM1_ESM.doc]

Table 1: Norms for the Enugu Summarization Scale

|  | Students | |  | |
| --- | --- | --- | --- | --- |
|  | Male | Female |  |  |
| Head  Mean Scores | 3.58 | 4.12 |  |  |
| Body:  Mean Scores  Std. Dev. | 7.22  4.40 | 7.73  2.81 |  |  |

***Validity***

The Scale has been found to correlate significantly with the Neurotic Illness Questionnaire developed in India, another developing country (Ebigbo et al, (1989).

E. Summary Evaluation

The author himself points to need for the collection of normative data for various groups or mentally and physically ill patients (Ebigbo et al, 1989).

**References**

Ebigbo. P.O (1982) Development of a Culture Specific (Nigeria) Screening Scale of Somatic Complaints Indicating Psychiatric Disturbance *Culture Medicine and Psychiatry, 6, 29-43.*

Ebigbo P.O. (1986) A Cross Sectional Study of Somatic Complaint of Nigerian Females Using the Enugu Summarization Scale Culture, *Medicine and psychiatry 10,167-186*

Ebigbo P.O Fanakiramaiah N. and Kumaraswamy N. (1989) Summarization in Cross Cultural Perspective, pp. 233-250. In Ebigbo O & Peltzer K (Ed) Clinical Psychology in Africa. Enugu: Chuka Printing Co. Ltd.

***** For further information and copies of Test Contact:**

Prof. P.O. Ebigbo.

Dept. of Mental Health,

College of Medicine

University of Nsukka.

**ESS**

AGE………………....……… SEX……..............….. OCCUPATION…………………………………

MARITAL STATUS……………………………….... FATHER’S OCCUPATION ……………………..

MOTHER’S LEVEL OF EDUCATION…………………………………………………..

**INSTRUCTION**

Please read each question carefully and tick Y for Yes and X for No on whether you agree with the statement(Y) or not(X) Endeavour to answer all the questions.

**HEAD**

1. Occasionally I experience heat sensation in my head
2. I have the feeling of something like water in my brain.
3. Sometimes it seems as if pepper were put into my head.
4. Things like ants keeps creeping in various parts of my brain.
5. I am convinced some types of worms are in my head.
6. If you look on my head exactly you can see it is sort of breathing.
7. Some spots/spot in my are so painful that I believe there is an injury or sore inside my brain.
8. My head seems to be bursting that I have to hold my head to prevent it.
9. I am convinced my head expands and contracts.
10. My head is so heavy that I feel I am carrying a heavy load.
11. I have very constantly severe headache.
12. By mere touching parts of my brain it hurts.
13. To be able to remain healthy; I must shave my hair completely and constantly.
14. My eyes are painful.
15. I can no longer see properly.
16. My eyelids are so heavy.
17. I have heat sensation on my eyes.
18. There are some hairs that seem to have entered my ear and blow some air constantly or make some constant noise.
19. For some time now I have been feeling very dizzy.
20. I have needle-like pinching in my head.
21. The beating in my head is like that from a hammer.
22. I have a feeling that something is blocking my throat.
23. When I swallow something, I can feel it travel very slowly down my throat to the stomach.

**BODY**

1. My shoulder is as heavy as if I were carrying a heavy load.
2. I have the feeling as if a 6-inch nail got stuck in my back.
3. I feel that there is some sore/injury on my chest, especially the left part of my chest.
4. I feel hot internally on all parts of my body.
5. I feel hot internally only on selected parts of my body.
6. Sometimes I have difficulty in breathing.
7. I breathe in such little air that I sometimes fear I would suffocate.
8. Intermittently I must breathe in fast otherwise I would suffocate.
9. Clearly parts of my body are out of order.
10. I know my body is not alright but nobody seems to believe me.
11. This my disorder appears to be out of reach of medical doctors.
12. I am convinced that only the traditional healers can do the job on me.
13. Such trouble as I have cannot easily be discovered by medical test.
14. Sometimes my heart suddenly wants to fly out (*Obi-ilo-mmiri*).
15. You can hear the beating of my heart from a distance.
16. I have biting sensation all over my body.
17. I feel pains right inside the marrow of the bones of my hands and legs.
18. Very often I sweat profusely without having done adequate physical exercise.
19. Something like worm live in my body crawling at times to different parts of the body at will.
20. The thing that worries me is not steady; it comes to different parts of the body at will.
21. I feel the various parts of my body shiver.
22. Sometimes I feel so restless that I fear I would not be able to control it and go mad.
23. It is difficult for me to explain to the doctor what is wrong with my body.
24. My problem is that I cannot sleep.
25. I feel general weakness on all parts of my body.
26. While walking my feet cannot stand firm on the ground.
27. Very often I have continuous noise in my belly.
28. My body is very is very light.
29. I have no erection at all in situation where I used to be sexually aroused.
30. I experience itching sensation on different parts of my body.
31. At the moment I get very weak erection.
32. My whole body is alright.
33. My whole body is dead: that is the feeling I have.
34. One part of my body is occasionally lamed.
35. Sometimes I get goose skin without warning.
36. My bodily symptom worsens after sexual intercourse.
37. I feel some minor cramps (Tita Ngweli).
38. I feel that I have dried up.
39. I feel pain each time I engage in sexual intercourse.
40. The middle of the sole to my feet is one of my main problems; I must continue to stretch my feet to get it somehow alright at times.
41. Whenever the sun is shining, I cannot walk far on foot, otherwise I am sure to collapse.
42. I feel a very heavy weight pressing me down whenever I sleep.
